# Validation of the Sensal Health MyAide^TM^ Smart Dock Medication Adherence Device

**DOI:** 10.3390/pharmacy13050123

**Published:** 2025-09-01

**Authors:** David Wallace, Sourab Ganna, Rajender R. Aparasu

**Affiliations:** 1 Department of Pharmacy Practice and Translational Research, University of Houston College of Pharmacy, Houston, TX 77204, USA; davewall@central.uh.edu; 2 Department of Pharmaceutical Health Outcomes and Policy, University of Houston College of Pharmacy, Houston, TX 77204, USA; sganna@central.uh.edu

**Keywords:** medical device, adherence tracking, compliance, medication adherence, medication dispensing systems

## Abstract

Background: Electronic monitoring adherence devices (EAMDs) are increasingly being utilized in various healthcare settings to track medication adherence. Objective: To determine the accuracy of the Sensal Health MyAide™ Smart Doc in capturing dose removal from the vial, specifically the time of dose removal and the number of pills removed for each actuation of the device. Methods: This validation study compares the device’s recording of dose withdrawals from a prescription vial by simulated patients against reference documentation reported using MS Forms by the participants. Three participants completed a 4-day study consisting of two non-consecutive 1 h sessions per day encompassing six actuations from the prescription vial to be captured by the Sensal Health MyAide™ Smart Dock after their informed consent was obtained. Statistical analysis included percent agreement and Cohen’s kappa assessing agreement between user-reported data and electronic measurement data recorded by the MyAide™ Smart Dock. Outcome measures included confirmation of the specific user, time of dose removal (±1 min), and the number of pills withdrawn. Results: Three subjects were recruited to provide data for a total of 144 actuations. The study found perfect 100% agreement across the number of pills withdrawn and specific users withdrawing the pills and 99% agreement for the time of administration. The Cohen’s kappa values for the outcome measures were 1.00 (95%CI [1.00, 1.00]) for the number of pills dispensed and specific user and 0.993 (95%CI [0.990, 0.996]) for the time of administration. Conclusions: This study found that the Sensal Health MyAide™ Smart Dock can accurately record the time of administration, the number of pills dispensed, and the identity of the user dispensing the pills.

## 1. Introduction

Poor medication adherence is associated with suboptimal treatment outcomes and increased healthcare costs. Medication adherence is commonly defined as the process by which patients take their medication as prescribed, composed of initiation and implementation [1]. The World Health Organization has documented that poor adherence to long-term therapies imperils the effectiveness of the therapies across a broad swath of health conditions. Their report showed how adherence impacted both health outcomes and economic costs [2]. More recently, a systematic review found that medication non-adherence across 14 disease groups resulted in between USD 949 and USD 52,341 in adjusted total costs [3].

Adherence can be measured through various methods, including patient self-report, pill counts, pharmacy refill data, biochemical assays, and electronic adherence monitoring devices (EAMDs), each with distinct advantages and limitations [4]. EAMDs are tools employed to improve medication adherence and are viewed as the “gold standard” for adherence measurement, particularly in measuring if a medication regimen has been implemented as prescribed [5]. In a meta-analysis of EAMDs, patients using EAMDs were found to have significantly better adherence when compared to the control when combined with a reminder and a health professional’s input [6].

EAMD technology includes inhaler monitors, electronic pill boxes, and electronic pill bottles [7,8,9] such as the Medication Event Monitoring System (MEMS) [6]. These devices record the date and time of each opening of the bottle. However, the number of doses removed per actuation cannot be captured by the system. International validation studies of EAMDs, such as those conducted in Europe and Asia, have reported high concordance between device-recorded data and reference standards, supporting their utility in clinical and research settings [10,11]. A study measuring non-adherence in kidney transplant recipients using the MEMS-5 Track Cap found that 62% of patients with reliable MEMS data had discrepancies between dispenses recorded and medication intake [12]. This study highlighted the challenges of measuring adherence using EAMDs only capable of capturing the opening of bottles. Furthermore, the study reported patients opening the bottles to demonstrate the device to a visitor, opening the wrong bottle, or taking the medication from another supply as causes for the difference [12].

Building on the capabilities of EAMDs, the integration of advanced technology like the Sensal Health MyAide™ Smart Dock into health system pharmacies has the potential to revolutionize medication management and adherence tracking. By accurately capturing not only the time of dose administration but also the exact number of pills dispensed, the MyAide™ Smart Dock provides detailed and reliable adherence data that goes beyond what traditional systems, like the Medication Event Monitoring System (MEMS), can offer. For health systems pharmacy, this has significant implications; pharmacists can leverage this real-time data to quickly identify patterns of non-adherence, allowing them to intervene early, adjust therapeutic regimens, and provide personalized counseling to improve patient outcomes. Furthermore, the ability of the MyAide™ Smart Dock to securely share data with healthcare teams enhances interdisciplinary collaboration, supports coordinated care efforts, and reduces the risk of medication errors. This level of monitoring and intervention can contribute to improved clinical outcomes, lower healthcare costs associated with non-adherence, and more efficient pharmacy operations, ultimately promoting better health system performance and patient care quality. This validation study was designed to determine the accuracy of the Sensal Health MyAide™ Smart Dock in capturing dose removal from the vial, specifically the time of dose removal, the number of pills removed for each actuation of the device, and whether the system can confirm that the designated person was responsible for the actuation.

## 2. Methods

### 2.1. MyAide™ Smart Dock and MyAide™ App

The MyAide™ Smart Dock is an electronic medication adherence device that captures the weight of the pill bottle and calculates the amount of solid oral medication dispensed per actuation. In tandem with providing a Near-Field Communication (NFC)-equipped pill bottle, the dock communicates and captures the removal from and replacement onto the dock of the medication bottle via weight measurement mechanisms, which was defined as an actuation. The removal and return of the NFC tag from the dock triggers the device to capture the date, time, and previous weight. The difference in weight before and after the actuation permits the number of dosage units removed to be calculated. Upon detection of an actuation, the MyAide™ Smart Dock, while communicating with the MyAide™ App, is designed to capture the timing of medication actuation and calculate the number of pills removed based on weight differences in the pill bottle. User input through the MyAide^TM^ app installed on the user’s mobile device is intended to serve as confirmation that the designated person has removed the medication. Although the app MyAide™ App can also generate personalized medication reminders and alerts to prompt patients for scheduled doses, these capabilities were not evaluated in the current study. Overall, the integration of the MyAide™ Smart Dock and MyAide™ App offers a user-friendly and technologically advanced approach to monitoring medication adherence and promoting patient engagement in medication adherence.

### 2.2. Study Design and Participants

This validation study compared the device’s recording of dose withdrawals from a prescription vial by participants against reference documentation reported by the participants using MS Forms. Small, hard-shelled mints (TicTac) were utilized as a substitute for oral tablets to minimize the risk of unnecessary exposure to an active medication by the study participants. Three subjects were recruited and enrolled in the validation study. Inclusion criteria of subjects were as follows: (1) active graduate students 18 years or older, (2) students possessing an iPhone capable of installing and running the MyAide™ Smart Dock MyAide™ App, and (3) availability to be physically present at the testing location during the training, run-in, and trial period. The exclusion criterion was as follows: (1) any students under the age of 18 years. This study followed the Strengthening the Reporting of Observational Studies in Epidemiology (STROBE) reporting guidelines.

The sample size calculation for the Cohen’s kappa agreement analysis was performed with the following parameters: expected kappa = 0.8, minimum acceptable kappa = 0.6, proportion of positive ratings = 0.5, alpha = 0.05, power = 0.80, and an anticipated dropout rate of 10%. This yielded a required number of 126 observations [13]. Our study achieved a total of 144 actuations, exceeding this threshold and thus providing sufficient power to assess agreement between the device and reference standard.

**Figure 1:**
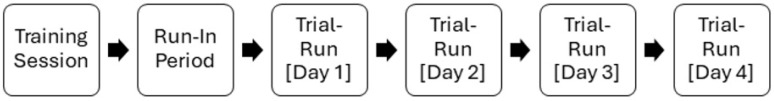
Timeline of study procedures.

Upon enrollment, the three subjects completed a training session where each subject was introduced to the MyAide™ Smart Dock device, the MyAide™ App, and Microsoft Forms, focusing on the usability, navigation, logging procedures, and overall testing procedures of future sessions. Each participant received 30 min, in-person training conducted by the study investigators, covering device operation, app use, Microsoft (MS) Forms logging, and procedural requirements for the run-in and trial phases. During the training sessions conducted by the authors, the subjects downloaded and installed the MyAide™ App on their respective mobile devices and were provided the login credentials for study accounts set up for their assigned MyAide™ Smart Docks. The study subjects were provided written step-by-step instructions for completing and documenting an actuation and were then asked to perform actuations in the presence of the first and second author to ensure that the MyAide™ Smart Dock, MyAide™ App, and MS Forms logs were all functioning properly. A run-in period was then performed to ensure that each subject was able to demonstrate all procedures involved in the study consistently without the study authors being immediately present. The run-in period consisted of 2 non-consecutive 1 h sessions on the same day. Lastly, a trial-run period was conducted encompassing 2 non-consecutive 1 h sessions per day for 4 days over 2 weeks (Figure 1). Short, non-consecutive sessions allowed for reduced fatigue in performing the protocol task by the study participants and allowed them to schedule the sessions around their other academic duties while also allowing for assessment of the device and its cloud-based support structure across multiple days.

**Figure 2:**
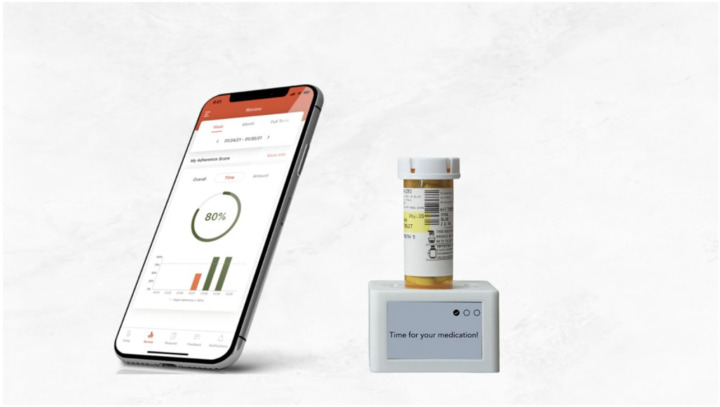
MyAide™ Smart Dock device and MyAide™ App.

Subjects were assigned a specific MyAide™ Smart Dock that was connected via Bluetooth to the MyAide™ App on the subjects’ phone with a provided user account to capture their adherence behavior (Figure 2). Each session required the subject to perform 6 actuations in 7 min intervals, where they would pull a prespecified number of pills (TicTac) from the pill bottle seated on the device. A 7 min interval for each actuation was selected (1) to ensure that the participant had adequate time to complete the steps required by the study protocol and (2) to provide a time period to identify distinct actuations with the logs. Each subject was also given a unique QR code linked to their respective dock to document the actuation on the MS Forms survey to log the subject’s adherence behaviors. Subjects were given access to the device to complete their sessions in a tightly controlled and secured space to ensure no distractions for all actuations and documentation steps throughout the study.

### 2.3. Outcomes

This validation study measured three outcomes that would confirm the specific user for each actuation, the specific time of actuation, and the number of pills withdrawn per actuation. Participants were instructed to interact with their MyAide™ App to tag each actuation they performed. The device data were obtained after three days of validation. The time stamp for this action, as compared to their MS Forms log entry, confirmed the identity of the person performing the actuation with an allowed window of 1 min. Lastly, the number of pills withdrawn during each actuation was documented in the MyAide™ App and MS Forms. The University of Houston Investigational Review Board approved the study.

### 2.4. Statistical Analysis

To assess the validation of the MyAide™ Smart Dock, an analysis was conducted using percent agreement, along with Cohen’s kappa, each followed by 95% confidence intervals. Interpretation of Cohen’s kappa was as follows: values 0.00–0.20 indicating no agreement, and <0.21–0.39 as minimal agreement, 0.40–0.59 as weak agreement, 0.60–0.79 as moderate agreement, 0.80–0.90 as strong agreement, and >0.90 as almost perfect agreement [14]. An alpha of 0.05 was used to determine statistical significance, and the collected data were analyzed using descriptive and inferential statistics with SAS version 9.4 [15].

## 3. Results

Three subjects were recruited and enrolled after informed consent was documented in writing. The three subjects completed all stages of the study: training day, run-in period, and trial run. A total of 144 actuations were documented at the conclusion of the 4-day period.

This study compared the data from the device against reference documentation reported by participants via MS Forms. Percent agreement and Cohen’s kappa were used to assess validation of the MyAide™ Smart Dock specifically to address dose removal and the number of pills removed for each actuation of the device. The analyses revealed 100% agreement for the number of pills dispensed and the validation of the specific dock responsible for dispensing pills, with a Cohen’s kappa coefficient of 1.00 (95% CI [1.00, 1.00]) (Table 1 and Table 2). Also, 99% agreement was found for the time of administration, with a Cohen’s kappa coefficient of 0.993 (95% CI [0.990, 0.996]).

## 4. Discussion

This validation study has demonstrated the robustness of the MyAide™ Smart Dock device in capturing real-time data on patient adherence behaviors. With perfect or near-perfect agreement observed across key outcome measures, including the number of pills dispensed, user identification, and timing of administration, our findings underscore the capabilities and effectiveness of this new technology in monitoring medication adherence. This capability to capture accurate and real-time adherence data is particularly advantageous in clinical settings where timely intervention is crucial for optimizing treatment outcomes. The utilization of the MyAide™ Smart Dock presents several strengths in enhancing medication adherence monitoring. Firstly, the integration of sensors within the MyAide™ Smart Dock enables accurate tracking of medication intake by detecting the removal and placement of medication bottles. This real-time data collection mechanism facilitates the timely recording of medication adherence events, allowing for comprehensive adherence monitoring. Additionally, the MyAide™ Smart Dock’s capability to calculate the quantity of pills consumed based on weight differences in the pill bottle adds another layer of accuracy to adherence measurement. Moreover, the MyAide™ Smart Dock combined with the MyAide™ App has the provision of personalized reminders and alerts to assist patients in adhering to their medication schedules, promoting better treatment adherence outcomes.

In addition to providing the time, the MyAide™ Smart Dock also provides information on the number of pills administered by the subject using weight-capturing technology. MEMS are a commonly used form of EAMD for tracking patient adherence, valued for their ability to record the exact times when medication bottles are opened. Medication adherence measured using MEMS and Self-Reported Questionnaires (SRQs) has been shown to be at least moderately correlated, suggesting that SRQs give a viable estimate of medication adherence [16]. However, the Sensal Health MyAide^TM^ Smart Dock offers advancements beyond MEMS. While MEMS primarily captures bottle openings, the MyAide™ Smart Dock provides a more accurate assessment of adherence by also calculating the number of pills taken based on weight differences. Furthermore, the MyAide^TM^ Smart Dock has the potential to offer seamless communication between patients, healthcare providers, and other care team members, enabling collaborative care efforts and timely intervention in cases of non-adherence. This advanced capability can significantly enhance adherence monitoring and improve clinical outcomes by ensuring that medication is not only accessed but consumed as prescribed.

While medication adherence and EAMDs are traditionally associated with regularly scheduled medications, as-needed (PRN) or titrated-to-effect therapies represent an important opportunity for these devices to help clinicians monitor patient response between clinic or office visits. A provider may prescribe a medication to a patient with instructions to adjust the dose taken based on criteria the patient can monitor and react to. For instance, a patient with hypertension who experiences volatility in their blood pressure may have a medication such as hydralazine added to their blood pressure regimen with instructions to take one tablet when their systolic pressure is closer to the goal and two tablets when the systolic pressure is above a threshold set by the clinician. An EAMD capable of measuring how many dosage units have been removed, such as the MyAide™ Smart Dock, can transmit that information to an online dashboard; the clinician could then monitor in real-time how frequently the patient was removing two tablets to address blood pressure readings above the threshold.

The ability for a provider to confirm that an actuation of an EAMD was performed by the intended person, patient, or caregiver is another capability that the MyAide™ Smart Dock brings when paired with the MyAide™ App. As our study has demonstrated, the study participant or patient can use the MyAide™ App and tag an actuation to indicate that they were the person removing the dose. This may be appealing to clinicians concerned about diversion in a participant’s or patient’s home when the medication involved either has a high potential for abuse or has a high monetary value.

The MyAide™ Smart Dock also brings the potential to measure adherence across several different dosage forms. While this study only simulated measuring the adherence of an oral solid dosage form, it is conceivable that the MyAide™ Smart Dock could have also been used to track the dosing of oral liquid medication, suppositories, or topical cream or ointment. If an NFC tag could be affixed to the medication container so the product rests with the tag centered on the dock, it should provide remote monitoring when the product is removed from the dock and how much was used during each actuation. Studies demonstrating this ability by the MyAide™ Smart Dock for other dosage forms or use in real-world settings remain to be published.

Compared to traditional systems such as the MEMS, which primarily capture the date and time a medication bottle is opened, the MyAide™ Smart Dock offers several notable advantages. Most significantly, it quantifies the number of pills dispensed during each actuation using weight-based measurements, enabling the detection of both under- and over-dosing—an important capability not available in MEMS. Additionally, the integration with the MyAide™ App allows for real-time user authentication, which can help address concerns related to medication diversion or unintentional administration by caregivers. Unlike MEMS, which is limited to solid oral dosage forms in standard pill bottles, the MyAide™ Smart Dock may be adapted for use with other dosage forms, such as oral liquids, topicals, or suppositories, through the application of NFC tags. These expanded functionalities enhance the utility of the device for broader clinical applications and support more accurate, personalized adherence monitoring.

One of the potential advantages of utilizing EAMDs is the ability to improve medication adherence among older adults, who may face challenges related to forgetfulness and cognitive decline [17,18,19]. EAMDs, along with the connected apps, can be valuable in providing reminders, alerts, and feedback mechanisms. These devices empower patients to adhere to their prescribed treatment regimens more effectively, thereby reducing the risk of medication errors and adverse events. Given proper development, adoption, and implementation, the ease of use and intuitive design of EAMDs make them suitable for older adults, enhancing their engagement in self-management and promoting autonomy in medication adherence. By leveraging connected devices and apps, EAMDs can advance patient-centered care delivery, improve treatment outcomes, and enhance the quality of life of individuals managing chronic conditions in real-world settings.

The implementation of EAMDs in clinical practice presents opportunities for further research and innovation in the field of medication adherence technology. Current medication tracking devices include smart pill dispensers and bottles, electronic pill organizers, wearable technology, and various other monitoring systems. These are a few examples of available products to aid in the care of a patient [11], all of which are poised with their nuanced strengths and weaknesses based on their mode of tracking and technological innovation and integration with supporting software. With the evolution of technology, along with cost-efficient manufacturing processes, innovative devices have scaled up in capability and applicability to a wide array of patients and disease states, providing the opportunity for wide-scale applications. Future studies focusing on evaluating the real-world effectiveness and sustainability of these devices in diverse patient populations and disease settings, as well as exploring novel approaches to enhancing device functionality, data analytics, and personalized interventions, are needed.

The integration of advanced EAMDs, such as smart docks and electronic organizers, offers significant potential to optimize medication management and adherence monitoring in the space of health systems pharmacy. With the ability to collect real-time data and share it seamlessly with healthcare teams, these technologies enable pharmacists to identify non-adherence trends early, intervene promptly, and personalize patient care plans. This can enhance patient outcomes, reduce medication errors, and minimize hospital readmissions, ultimately leading to cost savings for healthcare systems. Furthermore, these technologies allow clinicians and providers to work more efficiently within interdisciplinary teams, contributing to coordinated care efforts and ensuring that treatment goals are met across the continuum of care. Future studies should therefore focus not only on evaluating the real-world effectiveness and sustainability of these devices in diverse populations and disease settings but also on how these technologies can be fully integrated into health systems pharmacy to maximize their clinical and economic impact.

From a clinical implementation perspective, the MyAide™ Smart Dock holds potential for cost-effectiveness through its ability to reduce medication errors, improve adherence, and prevent costly hospitalizations or complications associated with non-adherence. Its scalability is supported by cloud-based data infrastructure and modular design, allowing deployment across diverse clinical settings. However, potential barriers to widespread adoption include the upfront cost of the device, the need for patient training to ensure proper use, and challenges in integrating adherence data with existing health systems. Addressing these barriers through streamlined onboarding processes, user-centered design, and interoperable data platforms will be essential to support broader clinical uptake.

## 5. Limitations

The major aim of this study was to independently validate the instrument’s ability to capture intended user actuations accurately and units removed during each actuation. The small study participant size (*n* = 3) of graduate students following detailed instructions in a controlled environment to eliminate confounding factors may limit the generalizability of the results to real-world settings. Additionally, a non-pharmaceutical product, TicTacs, was employed to reduce the risk of medication exposure to the participants. The use of TicTacs, in lieu of a product manufactured to USP standards, is acknowledged as a potential limitation to the application of the study results in clinical practice. Additionally, the study did not evaluate the ability of the instrument to distinguish the removal of dosage units with masses that differed substantially from those of the TicTac.

It is important to acknowledge other potential limitations, such as potential technical issues with the sensors or connectivity, that would impact the overall reliability of the data. Real-world issues such as variability in container sizes and shapes can present challenges in ensuring consistent weight measurements and accurate pill counts. Additionally, certain medication containers may require specific accommodations to be compatible with the Sensal Health MyAide™ Smart Dock, limiting its applicability. Patient acceptance and usability of the dock may also vary, potentially impacting adherence monitoring effectiveness in certain populations. Factors such as ease of use, the complexity of setup, and the need for regular maintenance or troubleshooting may pose barriers for some patients, particularly the elderly or those with limited technical proficiency. Addressing these challenges through a user-friendly design, complete technical support, and comprehensive patient education will be crucial in optimizing the effectiveness of the Sensal Health MyAide™ Smart Dock in real-world settings.

## 6. Conclusions

This validation study confirms the accuracy and reliability of the MyAide™ Smart Dock in tracking patient adherence behaviors in real-time, demonstrating near-perfect agreement across key metrics, including pill count, user identification, and administration timing. The MyAide™ Smart Dock utilizes advanced features like weight-capturing technology and real-time data synchronization, which enable precise medication intake monitoring and timely intervention opportunities. Although potential challenges in the real world remain, this technology shows promise in enhancing medication adherence within healthcare settings. The MyAide™ Smart Dock records events related to the removal of the pill bottle, but cannot confirm whether the removed medication was ingested. This distinction is important when interpreting adherence data, as non-ingestion after removal remains a potential source of concern in both research and clinical practice. Future research should explore the real-world effectiveness, sustainability, device functionality, data analytics capabilities, and development of personalized interventions to maximize the impact of EAMDs in clinical practice.

## Figures and Tables

**Table 1:** Percent Agreement—Percent Agreement for Key Measures Captured by the MyAide™ Smart Dock device.

Measures	Time of Actuation	Number of Pills Removed	Specific User per Actuation
Frequency of zeros	143	144	144
Count of observations	144	144	144
Percent agreement	99%	100%	100%

**Table 2:** Test Statistics (Cohen’s Kappa) for the Measures Assessed by the MyAide™ Smart Dock device.

Measure	Estimate	Standard Error	95% CI	
Time of actuation	0.993	0.002	0.990	0.996
Number of pills removed	1.000	0.000	1.000	1.000
Specific user per actuation	1.000	0.000	1.000	1.000

## Data Availability

Data can be shared upon reasonable request.

## References

[1] Vrijens B., De Geest S., Hughes D.A., Przemyslaw K., Demonceau J., Ruppar T., Dobbels F., Fargher E., Morrison V., Lewek P. (2012). A new taxonomy for describing and defining adherence to medications. Br. J. Clin. Pharmacol..

[2] Chaudri N.A. (2004). Adherence to Long-term Therapies Evidence for Action. Ann. Saudi Med..

[3] Cutler R.L., Fernandez-Llimos F., Frommer M., Benrimoj C., Garcia-Cardenas V. (2018). Economic impact of medication non-adherence by disease groups: A systematic review. BMJ Open.

[4] Lam W.Y., Fresco P. (2015). Medication Adherence Measures: An Overview. BioMed Res. Int..

[5] Vrijens B., Antoniou S., Burnier M., de la Sierra A., Volpe M. (2017). Current Situation of Medication Adherence in Hypertension. Front. Pharmacol..

[6] Chan A.H.Y., Foot H., Pearce C.J., Horne R., Foster J.M., Harrison J. (2022). Effect of electronic adherence monitoring on adherence and outcomes in chronic conditions: A systematic review and meta-analysis. PLoS ONE.

[7] Apter A.J., Tor M., Feldman H.I. (2001). Testing the reliability of old and new features of a new electronic monitor for metered dose inhalers. Ann. Allergy Asthma Immunol. Off. Publ. Am. Coll. Allergy Asthma Immunol..

[8] Burgess S.W., Wilson S.S.I., Cooper D.M., Sly P.D., Devadason S.G. (2006). In vitro evaluation of an asthma dosing device: The smart-inhaler. Respir. Med..

[9] McGrady M.E., Ramsey R.R. (2020). Using Electronic Monitoring Devices to Assess Medication Adherence: A Research Methods Framework. J. Gen. Intern. Med..

[10] Arnet I., Rothen J.P., Hersberger K.E. (2019). Validation of a Novel Electronic Device for Medication Adherence Monitoring of Ambulatory Patients. Pharmacy.

[11] Mason M., Cho Y., Rayo J., Gong Y., Harris M., Jiang Y. (2022). Technologies for Medication Adherence Monitoring and Technology Assessment Criteria: Narrative Review. JMIR mHealth uHealth.

[12] Denhaerynck K., Schäfer-Keller P., Young J., Steiger J., Bock A., De Geest S. (2008). Examining assumptions regarding valid electronic monitoring of medication therapy: Development of a validation framework and its application on a European sample of kidney transplant patients. BMC Med. Res. Methodol..

[13] Arifin W.N. (2025). Sample Size Calculator (Web). http://wnarifin.github.io.

[14] McHugh M.L. (2012). Interrater reliability: The kappa statistic. Biochem. Medica.

[15] SAS Help Center: SAS/STAT User’s Guide: High-Performance Procedures. https://documentation.sas.com/doc/en/pgmsascdc/9.4_3.5/stathpug/titlepage.htm.

[16] Shi L., Liu J., Fonseca V., Walker P., Kalsekar A., Pawaskar M. (2010). Correlation between adherence rates measured by MEMS and self-reported questionnaires: A meta-analysis. Health Qual. Life Outcomes.

[17] Punnapurath S., Vijayakumar P., Platty P.L., Krishna S., Thomas T. (2021). A study of medication compliance in geriatric patients with chronic illness. J. Fam. Med. Prim. Care.

[18] Bastani P., Bikineh P., Ravangard R., Rezaee R., Kavosi Z. (2021). Determinants affecting medication adherence in the elderly: A qualitative study. Aging Med. Milton NSW.

[19] Rodgers J.E., Thudium E.M., Beyhaghi H., Sueta C.A., Alburikan K.A., Kucharska-Newton A.M., Chang P.P., Stearns S.C. (2018). Predictors of Medication Adherence in the Elderly: The Role of Mental Health. Med. Care Res. Rev. MCRR.

